# Supplementary figures and images for: Porcine Sapovirus Protease Controls the Innate Immune Response and Targets TBK1
Source: Viruses. 2024 Feb 3;16(2):247. doi: 10.3390/v16020247 (PMC10892870; doi:10.3390/v16020247)

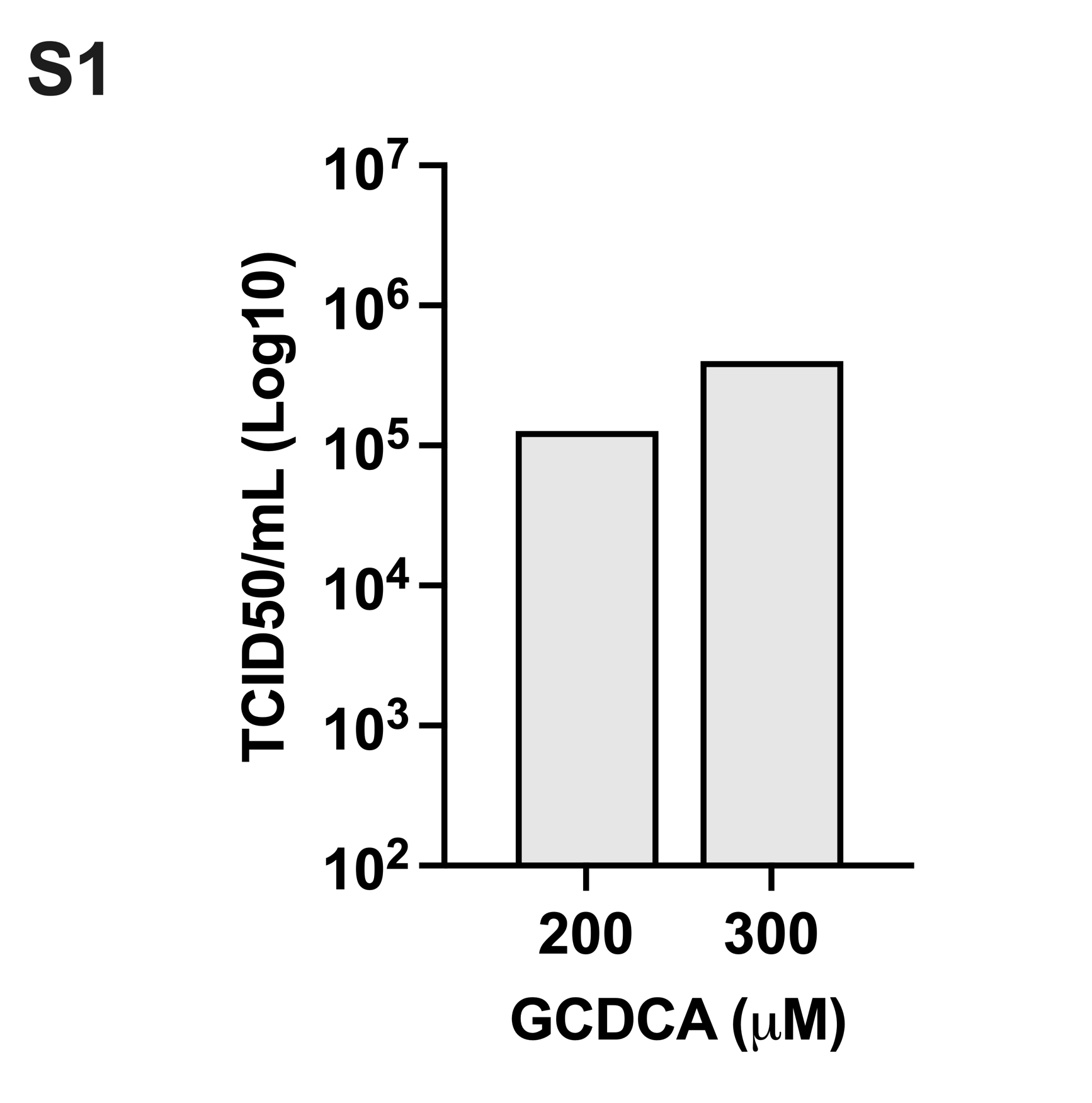

Supplement: Supplementary file 1 [file viruses-16-00247-s001.zip › Figure S1.tiff]

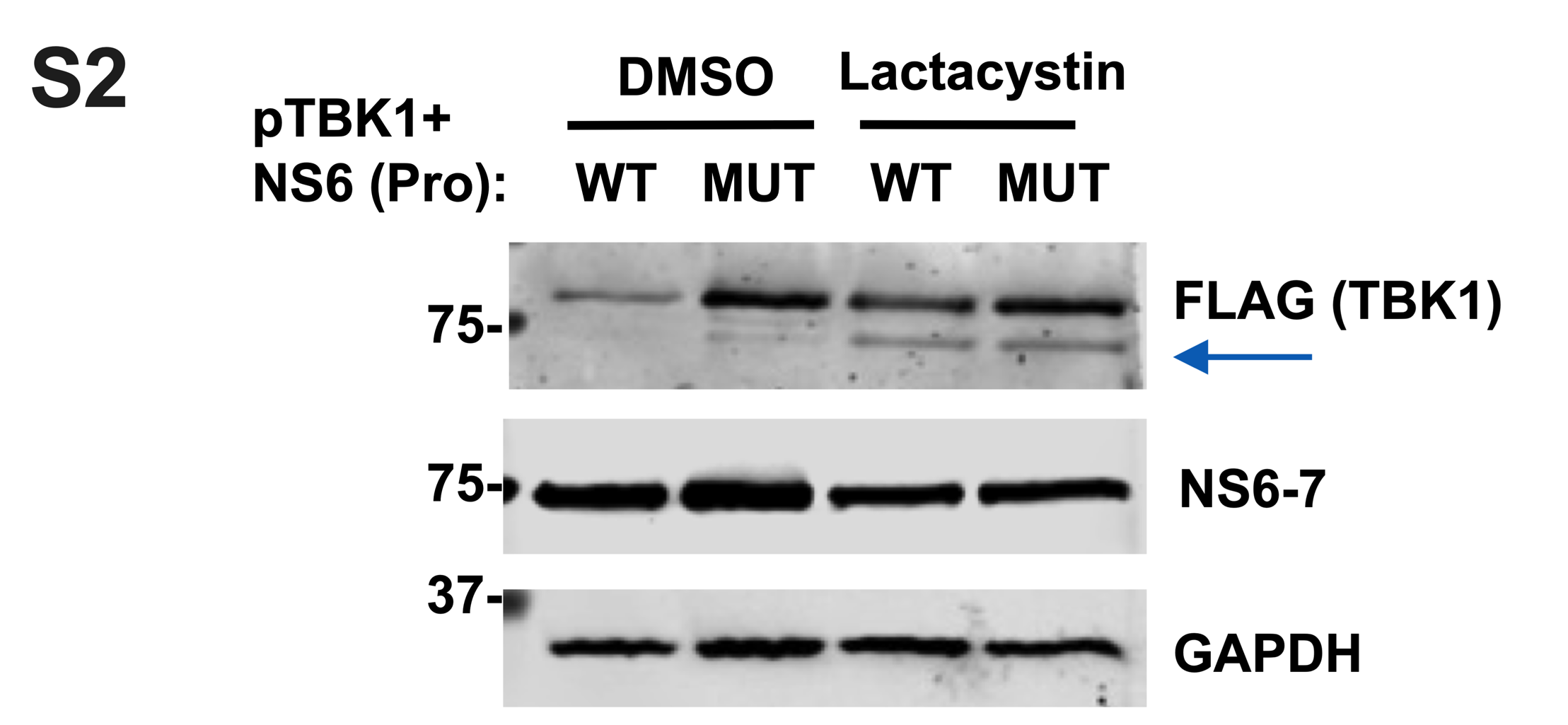

Supplement: Supplementary file 1 [file viruses-16-00247-s001.zip › Figure S2.tiff]

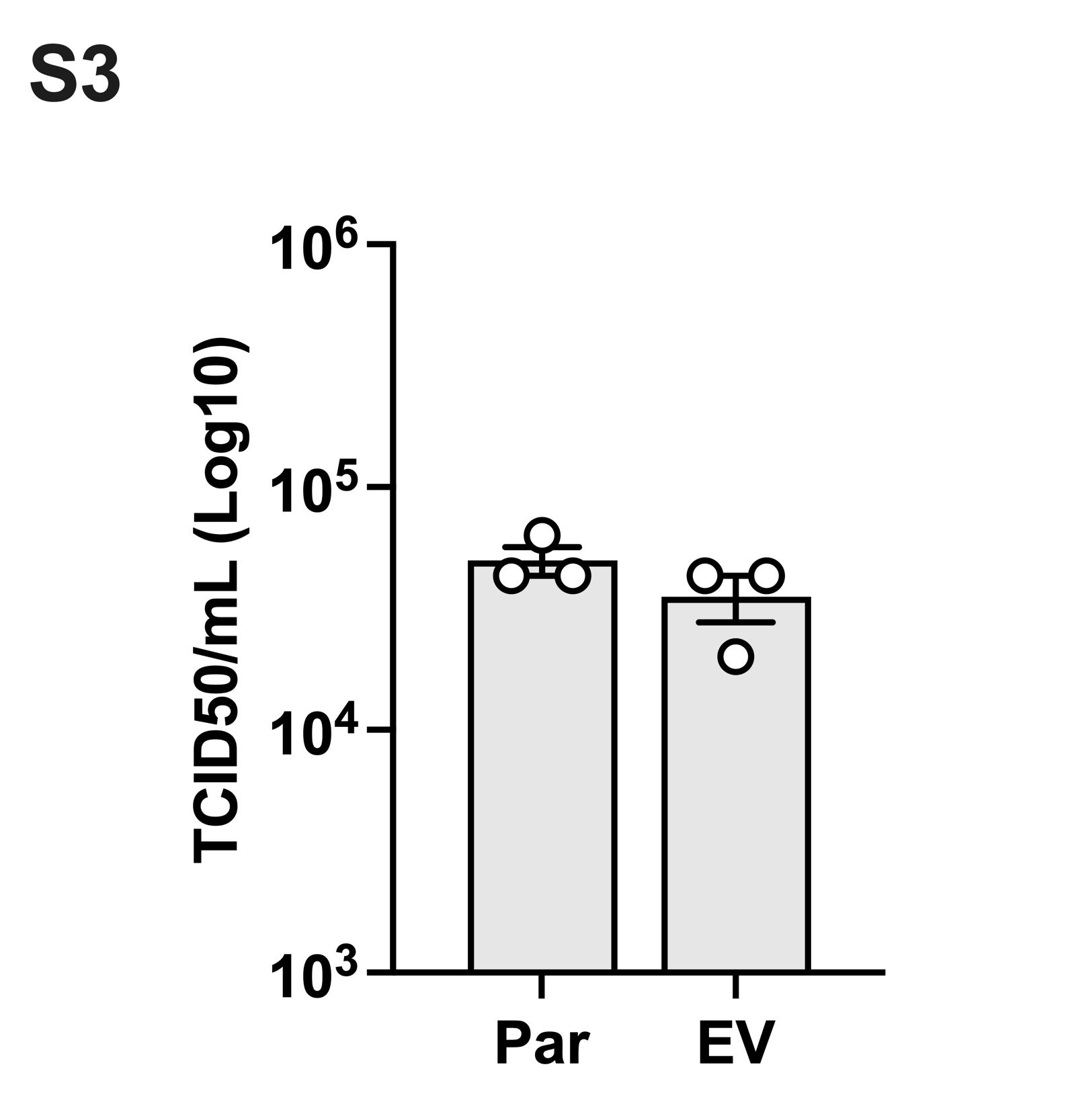

Supplement: Supplementary file 1 [file viruses-16-00247-s001.zip › Figure S3.tiff]
